# Self-management Interventions for People With Parkinson Disease: Scoping Review

**DOI:** 10.2196/40181

**Published:** 2022-08-05

**Authors:** Madison Milne-Ives, Camille Carroll, Edward Meinert

**Affiliations:** 1 Centre for Health Technology University of Plymouth Plymouth United Kingdom; 2 Peninsula Medical School Faculty of Health University of Plymouth Plymouth United Kingdom; 3 Department of Primary Care and Public Health School of Public Health Imperial College London London United Kingdom; 4 Harvard TH Chan School of Public Health Harvard University Boston, MA United States

**Keywords:** Parkinson disease, self-management, self-care, home nursing, self-efficacy, quality of life, signs and symptoms, health behaviour

## Abstract

**Background:**

Parkinson disease can impose substantial distress and costs on patients, their families and caregivers, and health care systems. To address these burdens for families and health care systems, there is a need to better support patient self-management. To achieve this, an overview of the current state of the literature on self-management is needed to identify what is being done, how well it is working, and what might be missing.

**Objective:**

The aim of this scoping review was to provide an overview of the current body of research on self-management interventions for people with Parkinson disease and identify any knowledge gaps.

**Methods:**

The PRISMA-ScR (Preferred Reporting Items for Systematic Reviews and Meta-Analyses Extension for Scoping Reviews) and Population, Intervention, Comparator, Outcome, and Study type frameworks were used to structure the methodology of the review. Due to time and resource constraints, 1 reviewer systematically searched 4 databases (PubMed, Ovid, Scopus, and Web of Science) for the evaluations of self-management interventions for Parkinson disease published in English. The references were screened using the EndNote X9 citation management software, titles and abstracts were manually reviewed, and studies were selected for inclusion based on the eligibility criteria. Data were extracted into a pre-established form and synthesized in a descriptive analysis.

**Results:**

There was variation among the studies on study design, sample size, intervention type, and outcomes measured. The randomized controlled trials had the strongest evidence of effectiveness: 5 out of 8 randomized controlled trials found a significant difference between groups favoring the intervention on their primary outcome, and the remaining 3 had significant effects on at least some of the secondary outcomes. The 2 interventions included in the review that targeted mental health outcomes both found significant changes over time, and the 3 algorithms evaluated performed well. The remaining studies examined patient perceptions, acceptability, and cost-effectiveness and found generally positive results.

**Conclusions:**

This scoping review identified a wide variety of interventions designed to support various aspects of self-management for people with Parkinson disease. The studies all generally reported positive results, and although the strength of the evidence varied, it suggests that self-management interventions are promising for improving the care and outcomes of people with Parkinson disease. However, the research tended to focus on the motor aspects of Parkinson disease, with few nonmotor or holistic interventions, and there was a lack of evaluation of cost-effectiveness. This research will be important to providing self-management interventions that meet the varied and diverse needs of people with Parkinson disease and determining which interventions are worth promoting for widespread adoption.

## Introduction

### Background

Parkinson disease has a substantial impact on patients, their caregivers and families, and health care systems globally [1,2]. The United Kingdom’s aging population is expected to nearly double the prevalence of Parkinson disease by 2065 [3]. The National Health Service (NHS) Long Term Plan has emphasized the need for supported self-management to improve patient outcomes and reduce the strain of an aging population on the health care system [4]. Self-management support interventions for chronic illnesses have been demonstrated to decrease health care use without negatively affecting patient health outcomes [5], but research is still needed on how they are used by and affect all users (including patients, caregivers, and health care professionals) [6]. The NHS has estimated that 25% to 40% of patients have low self-management knowledge, skills, and confidence (patient activation) [7]. A recent study focusing on people with Parkinson disease found that more than half of the patients rated themselves high on patient activation, whereas perceived self-management support was rated much lower [8].

Although Parkinson disease itself is not fatal, its complications and motor and nonmotor symptoms can have serious negative effects on the quality of life for both patients and care partners (CPs). The nonmotor symptoms of Parkinson disease are often undeclared in routine appointments [9] but can have severe negative effects on symptom burden and the quality of life [10,11]. For instance, it has been estimated that around half of the people with Parkinson disease have a mental health comorbidity [12]. Parkinson disease has a substantial impact on patients, their CPs and families, and health care systems [1,13,14]. Successful self-management is associated with improvements in chronic conditions and achieved by supported self-efficacy [15]. For all Parkinson disease symptoms, there are pharmacological and nonpharmacological approaches to management. Self-management interventions focus on the nonpharmacological approaches to symptoms by providing people with Parkinson disease and CPs with support to identify and monitor their symptoms and behavioral approaches to manage their symptoms [16,17].

### Preliminary Literature Review

Previous systematic reviews have examined various aspects of support for people with Parkinson disease, particularly interventions that support a shift toward more home-based care, but none were identified that provided a comprehensive overview of self-management interventions. There are 2 recent systematic reviews that examined the use of digital technologies and wearables to monitor or support the care of people with Parkinson disease and provide a comprehensive and recent overview of the available technologies, what they are being used for, and how they are being evaluated [18,19]. A recent preprint review provided an overview of the trends in research in the use of mobile and wearable technology for Parkinson disease over the past decade and identified 4 main applications: assisting with diagnosis, monitoring and prognosis, predicting the outcomes of treatments, and therapy [20]. A scoping review conducted in 2018 summarized the literature about home-based rehabilitation interventions [21].

There were 2 reviews that focused specifically on self-management for people with Parkinson disease [22,23]. The first was a systematic review of the qualitative experience of self-management components by people with Parkinson disease and their carers [22]. This review identified 7 key aspects of self-management interventions for people with Parkinson disease: “(1) medication management, (2) physical exercise, (3) self-monitoring techniques, (4) psychological strategies, (5) maintaining independence, (6) encouraging social engagement, and (7) providing knowledge and information” [22]. However, it did not provide an overview or evaluation of the impact of the self-management interventions on health, behavioral, or other outcomes. The other review was an integrative literature review, which provided an overview of the characteristics of self-management support programs for people with Parkinson disease and their effectiveness [23]. It identified a wide variety of interventions, most of which were specific to Parkinson disease, but found limited evidence of their effectiveness. The review provided a good summary of the state of the field but was conducted in 2016 and did not examine the integration of digital technologies in self-management interventions.

### Rationale

A search of the international prospective register of systematic reviews (PROSPERO) also did not find any relevant reviews on self-management and Parkinson disease in progress. A search for “parkinson AND (digital OR technolog*) AND (self-management OR home based care)” only retrieved 4 registrations: 1 focused on diabetes, and the others included a range of neurological conditions. A broader search for “parkinson AND self-management” identified 1 relevant registration—a systematic review and meta-analysis of self-management interventions in Parkinson disease. However, the registration is 2 years old (published on PROSPERO on April 15, 2019), has not been updated, and was not identified in a search for a published final article [24].

Given the rapid evolution of digital technology [25] and its growing role in health care [4], the state of the literature on self-management interventions has likely changed since the 2016 review was conducted, necessitating an updated overview that intentionally includes digital interventions, which are becoming a desired support for Parkinson disease care [26]. The variety of self-management aspects and applications identified in previous reviews indicates that an overview of the different types of self-management interventions and their potential impact is needed. The needs emphasized by the NHS Long Term Plan [4] for self-management and technology-enabled, personalized care demonstrate the potential for digital technology to help people with Parkinson disease and CPs improve their identification and management of Parkinson disease symptoms. Understanding the types of self-management interventions currently being developed and implemented will help inform the development of future, digitally enabled self-management interventions.

### Objectives and Research Questions

The aim of this scoping review was to provide an overview of the current state of the field and the evidence of the effectiveness of self-management interventions for Parkinson disease and to identify any gaps. Specifically, the review asked, “What types of self-management interventions are available to support people with Parkinson disease, what outcomes do they target, and what evidence is there in the literature of their effectiveness?”

## Methods

### Search Strategy

The PRSIMA-ScR (Preferred Reporting Items for Systematic Reviews and Meta-Analyses Extension for Scoping Reviews; Multimedia Appendix 1 [27]) and Population, Intervention, Comparator, Outcome, and Studies (PICOS) frameworks were used to structure the review and develop the search strategy (see Table 1). Based on the PICOS, relevant Medical Subject Headings (MeSH) terms were identified from a preliminary search, and the search string was created using the following structure: Population (MeSH terms) AND Interventions (MeSH terms) AND Outcomes (MeSH terms). There was no limit on the publication date. The search was performed in 4 databases—PubMed, Ovid, Scopus, and Web of Science—using the University of Plymouth’s search tool Primo. PubMed was chosen because it provides a good synthesis of biomedical literature, and the other search engines were selected because they capture a broad, multidisciplinary set of databases to ensure that no relevant literature was missed. Multimedia Appendix 2 provides a complete record of the specific search strings (modified slightly to fit the specific structure and requirements of each database) and the number of references retrieved. The database searches were performed on April 8, 2021.

**Table 1:** PICOS framework.

PICOS^a^	Detail	MeSH^b^ terms used in search
Population	People with Parkinson disease and their carers	Parkinson Disease
Intervention	Self-management interventions for people with Parkinson disease	Self-Management OR Self-Care OR Home Nursing OR Delivery of Health Care, Integrated OR Telemedicine OR Mobile Applications OR Internet-based Interventions OR Internet of Things
Comparator	None or standard care	—^c^
Outcomes	Primary outcome: self-management (with measures including, but not limited to, health outcomes, behaviors, perceived self-efficacy, quality of life, and use of health care services, etc) Secondary outcomes: factors that could affect self-management (eg, demographics and disease factors, etc)	Self Efficacy OR Quality of Life OR Signs and Symptoms OR Health Behaviour OR Patient Admission OR Patient Readmission
Study types	Case-control studies, cohort studies, and RCTs^d^	—

^a^PICOS: Population, Intervention, Comparator, Outcome, Study type.

^b^MeSH: Medical Subject Headings.

^c^Not applicable.

^d^RCT: randomized controlled trial.

### Inclusion Criteria

Studies were eligible for inclusion in the review if they evaluated a self-management intervention for people with Parkinson disease or their CPs. A broad definition of self-management interventions was used, so that an overview of the different types of intervention could be collected. Any intervention type (remote or in person) was eligible for inclusion if it aimed to help improve any elements of the patient self-management of Parkinson disease. Randomized controlled trials (RCTs), cohort studies, and case-control studies were eligible for inclusion. Studies published at any date were eligible for inclusion.

### Exclusion Criteria

Studies were excluded if they did not include a self-management intervention for Parkinson disease or if they described an intervention without evaluating it. Protocols and reviews were also excluded. Studies that were published in languages other than English were also excluded, as the review team did not have the necessary resources to assess them.

### Screening and Article Selection

**Figure 1:**
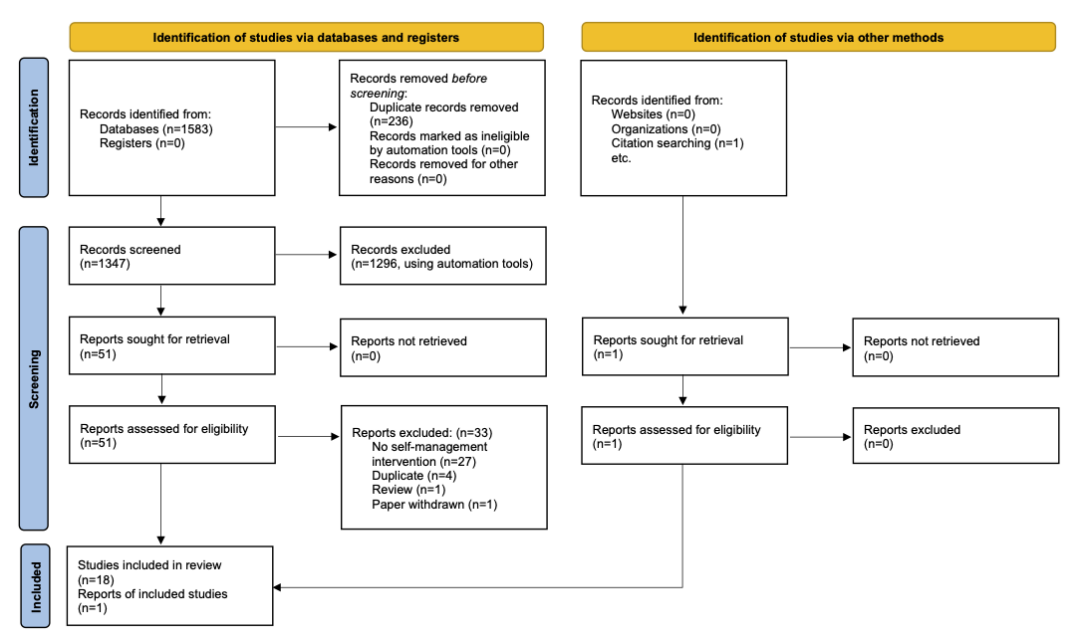
PRIMSA (Preferred Reporting Items for Systematic Reviews and Meta-Analyses) flow diagram.

The EndNote X9 citation management software (Clarivate) was used to store references, remove duplicates, and conduct the initial screening. The screening was done in several stages, using keywords based on the PICOS (see Multimedia Appendix 3). Next, 1 reviewer screened the remaining titles and abstracts (excluding articles with reasons) and conducted a full-text review to determine final eligibility (see Figure 1). The review team did not have the resources—in terms of time and budget—to have a second reviewer screen and extract data.

### Data Extraction

The full texts of the included studies were reviewed, and the data were extracted by 1 reviewer based on a predetermined form (see Textbox 1). Given the anticipated variety of study types and aims, the specific outcomes to extract were not prespecified but included as data to be extracted.

**Article information and data extraction.**

**General study information**

- Title
- Year of publication
- Sample size
- Population
- Method

**Intervention**

- Type of self-management intervention
- Description of self-management intervention

**Evaluation**

- Primary outcome
- Secondary outcomes
- Summary of reported results
- Evidence of effectiveness at achieving stated outcomes

### Data Analysis and Synthesis

A descriptive analysis was used to summarize the data extracted from the studies and provide an overview of the state of the literature on self-management interventions for Parkinson disease. The implications of the findings are examined in the discussion.

## Results

### Included Studies

The database search retrieved 1583 references (see Multimedia Appendix 2). The EndNote X9 software was used to remove 236 duplicates, and the keyword search tool was used to screen out 1296 references (see Multimedia Appendix 3). The titles and abstracts of 51 studies were screened by 1 reviewer, and articles were excluded with reasons. Of these 51 articles, 19 were selected for the full-text review. Subsequently, 1 study was identified as a duplicate upon full-text review [28], and 1 study was a secondary data analysis of an RCT, so the original RCT was identified and included [29], resulting in a final set of 19 included studies. The reasons for exclusion in the full-text review stage are detailed in Figure 1. The table with the extracted data is included as Multimedia Appendix 4.

### Study Characteristics

The largest proportion (8/19, 42% or 7/18, 39%, as 2 of the studies referred to the same trial) of the studies included in the review used an RCT methodology [29-36]. The remaining studies used a variety of study types, including 3 feasibility studies evaluating algorithms [37-39], 2 one-arm pre-post trials [40,41], 2 pilot studies [42,43], 2 mixed methods acceptability studies [44,45], a randomized case-control study [46], and a secondary data analysis of program adherence [47].

The studies also had a wide range of sample sizes, from 11 participants [42] to 474 participants [31]. All of the 8 studies with the largest sample sizes (greater than 100 participants) were RCTs [29-36].

**Figure 2:**
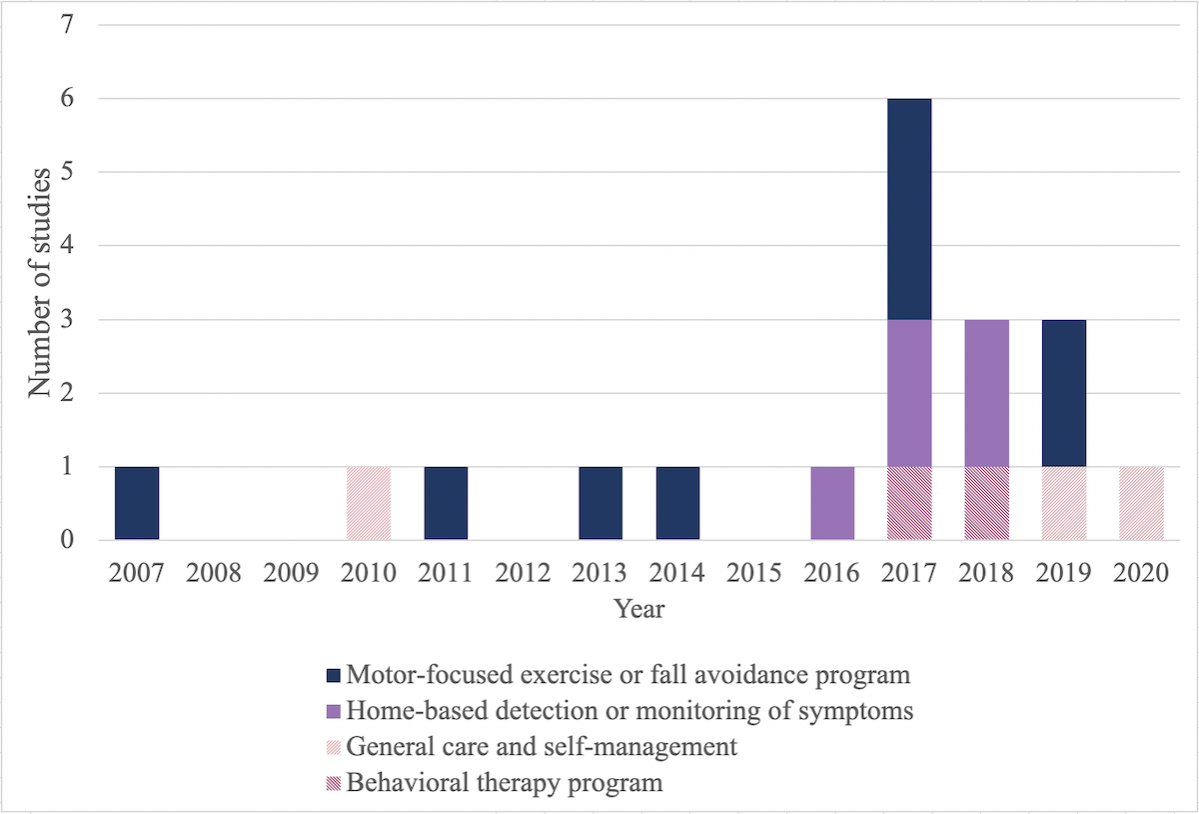
Number of included studies by year and category of Parkinson disease self-management intervention. The intervention types included home-based detection or monitoring of symptoms [37-39,44,46], general care and self-management [29,35,45], behavioral therapy program [40,43], and motor-focused exercise or fall avoidance program [30-34,36,41,42,47]. Note that 2 of the papers included in general care and self-management relate to the same study [29,45].

The earliest study included in the review was published in 2007 [30] and the latest in 2020 [45], but two-thirds (13/19, 68%) of the studies were published in 2017 or later (see Figure 2).

### Types of Interventions

A variety of different types of self-management intervention were described and evaluated by the included studies. The most common (9/19, 47%) type of intervention was home-based exercise or fall prevention programs [30-34,36,41,42,47]; within this category, two-thirds (6/9, 67%) were motor-related exercises or fall avoidance programs, and the remaining interventions were 1 that used sensor-based feedback [42], a community-based exercise program [32], and a handwriting program [33]. Several (5/19, 26%) interventions provided a means of home-based detection or monitoring of symptoms, which were also primarily focused on motor symptoms [37-39,44,46]. Of the remaining studies, 2 delivered behavioral therapy–type interventions to address mental health outcomes [40,43], 1 (addressed in 2 papers) delivered a nurse-led care management program [29,45], and 1 examined a rehabilitation program specifically focusing on self-management skills [35].

### Evidence of Effectiveness

#### Summary

A variety of different outcome measures were used by the studies to evaluate the interventions, given their different aims and types. The strongest evidence of effectiveness came from the 8 RCTs [29-36]. In all, 5 of the 8 RCTs found significant evidence that the intervention was more effective than the control at achieving its respective primary outcome: a 2-minute walk [32], reducing fall rates [30], self-perceived performance of daily activities [34], motor score on the Movement Disorders Society–Unified Parkinson's Disease Rating Scale [36], and health-related quality of life [35]. The other 3 RCTs [29,31,33], a pre-post trial [41], and 1 of the pilot studies [42] found significant differences (between groups or over time) for some, but not all, of the outcomes measured.

#### Motor-Focused Program Outcomes

Several of the studies examined interventions that included sessions with physiotherapists or occupational therapists combined with independent practice aimed at preventing falls or improving physical activity function [30,31,34,41]. Of the 2 studies that focused specifically on fall prevention, 1 found a significantly reduced rate of falls in the intervention group compared to the control group [30]. The other study (PDSAFE personalized fall prevention program) did not find a significant difference in repeated falling between groups but did observe better balance, functional strength, and fall efficacy and reduced near-falls in the intervention group compared to the control group [31]. The remaining 2 studies focused on exercise; 1 found significant improvements in outcome expectations for exercise and time spent exercising and on the Unified Parkinson’s Disease Rating Scale and the Parkinson’s Disease Questionnaire-39 but not self-efficacy, outcome expectations for functional ability, depression, or timed chair rise scores [41], whereas the other found a significant difference on self-perceived performance in daily activity but not perceived capacity, daily activity performance, effect of fatigue, coping skills, mood, or quality of life measures [34].

There were 2 studies of motor-focused interventions that used digital technology to provide self-management support [36,42]. These studies found that a virtual reality home-trainer stationary cycle resulted in a significant difference between intervention and control groups on the Unified Parkinson’s Disease Rating Scale in favor of the intervention [36] and that a sensor-based auditory feedback device to improve stepping automaticity while dual-tasking had a significant difference for step automaticity but not for fear of falling, cognitive functioning, or self-reported gait freezing [42].

The remaining 2 studies of the motor-focused interventions delivered a community exercise intervention with aerobic and resistance training twice weekly, which found significant effects for 2-minute walk scores and the Unified Parkinson’s Disease Rating Scale over 12 months [32], and a control intervention focused on handwriting, which found some effect on self-reported difficulty [33].

#### Mental Health Outcomes

There were 2 studies that examined the impact of interventions on mental health outcomes related to Parkinson disease [40,43]. Both trials examined the effect of an intervention over time and found significant improvements with large effect sizes. One of the studies, which was pilot-testing a 10-week cognitive-behavioral telemedicine program (a self-help workbook combined with occasional telephone sessions), found a significant improvement in depression and anxiety over the 4-month study period [43]. The other study, which evaluated a 6-week telephone-based behavioral activation intervention, found a significant, medium-to-large effect size of the intervention on apathy (*d*=0.77), depression (*d*=0.70), and quality of life (*d*=0.50) [40].

#### Algorithm Evaluations

There were 2 studies that evaluated a classification algorithm by measuring area under the receiving operator curve (AUC) as their primary outcome. The AUC represents how well the model can differentiate between 2 conditions, with a general understanding that scores of 0.7-0.8 are acceptable, 0.8-0.9 are excellent, and 0.9 and higher are outstanding [48]. The first study found AUCs of 0.88 and 0.91 for the best models [37], and the other validated that the model performed similarly on data collected in clinic (AUC 0.83) as on data collected at home (AUC 0.76) [38]. A third study evaluating an algorithm reported that it compared favorably to similar systems and that it could replicate clinical decisions; however, the supervised machine learning process was based on the decisions of 1 neurologist, so the algorithm had learned to replicate those decisions [39]. The authors recognized this as a limitation of the study.

#### Patient Perceptions, Acceptability, and Usability

Of the 19 studies, 2 used mixed methods to examine the user perceptions of the interventions [44,45]. The first was a companion study to a multisite RCT of a nurse-led care management intervention for Parkinson disease [29]. The study found that after the intervention, people with Parkinson disease rated their medication self-management highly and found the nurse care managers to be helpful, although some usability issues with the program were reported by participants and nurse care managers. Likewise, the nurses found the program to be helpful, the Parkinson disease specialists found the nurse care manager’s role to be helpful, and both reported seeing improvements in the self-management of people with Parkinson disease [45].

The other study assessed the acceptability of a wrist-worn sensor [44]. Participants identified discomfort after long periods of use and problems with the strap; however, there was high compliance with wearing the sensor, and participants reported a preference for the sensor over symptom diaries [44].

#### Adherence

There was 1 article [47] that reported a secondary data analysis for 1 of the RCTs included in the review [30]. It examined the adherence of the 70 participants in the intervention group of the study to the home-based exercise program. Patients reported completing a high percentage (79%) of the recommended number of repetitions of their exercises. Adherence varied depending on participant characteristics; specifically, older age, worse physical condition, pain, anxiety, and depression were all associated with reduced adherence to the prescribed exercises [47].

#### Cost-effectiveness

Only 1 study focused on assessing cost-effectiveness [46]. Cubo et al [46] conducted a randomized, case-control study comparing home-based motor monitoring (using wireless motion sensors) with in-office monitoring. They reported that the home-based monitoring was cost-effective but found no significant differences between the groups for symptoms or quality of life [46].

## Discussion

### Summary of Findings

The studies included in the review varied widely in terms of the study and intervention types, the number of participants, and the outcomes assessed. Approximately two-thirds (13/19) of the studies included examined interventions that focused primarily on motor-related outcomes. Almost 40% (7/18) of the studies included were RCTs; these trials had the largest sample sizes (ranging from 105 to 474 participants) compared to the other study types (ranging from 11 to 82 participants).

The RCTs also had the strongest evidence of effectiveness for their interventions, with almost two-thirds (5/8) finding significant evidence of effectiveness for their primary outcome compared to the control group, and the remaining 3 studies finding significant evidence for some of their outcome variables. However, the non-RCT studies all had at least some evidence to support their intervention, including evidence of an effect of the intervention over time, good model fit, adherence, generally positive acceptability and user perceptions, or cost-effectiveness. However, several of these studies reported limitations in their design and emphasized the need for further investigations to address unanswered questions.

### Limitations

A limitation of this scoping review is that only 1 researcher performed the article selection, data extraction, and data analysis. The PRISMA-ScR framework was used to guide the review [27] and ensure that the requirements for a scoping review were reported, but we could not prevent any potential bias due to the lack of validation from a second, independent reviewer.

Another potential source of selection bias is that no manual searches were conducted in the references of any of the included articles or reviews retrieved in the initial search. Due to time constraints, this search was not feasible but increases the possibility that relevant studies may not have been included in the review.

### Meaning and Future Research

The volume of studies retrieved during the initial search and the variety of intervention types included in the review demonstrate the breadth of research on technological support and home-based care for Parkinson disease. The research into supporting self-management for people with Parkinson disease addressed several different aspects of management: home-based symptom monitoring that aimed to improve data collection and better inform health care professionals’ care decisions; behavioral therapy that aimed to improve mental health; and independent, supervised, or community programs that aimed to increase mobility and strength, reduce the risk of falls, and improve the quality of life.

In addition to the variety of the research, this review identified some trends in the interventions being developed and evaluated to support self-management in people with Parkinson disease. The most prominent trend was the focus of the interventions on motor-related monitoring and care. This was an interesting observation, because the nonmotor symptoms of Parkinson disease can have a substantial impact on disease burden and the quality of life [49-52]. Only 2 of the reviewed interventions focused primarily on the mental health aspects of Parkinson disease [40,43]. Although some (6/17) of the other studies did include an assessment of at least one nonmotor symptom as a secondary outcome (most frequently depression) [29,31,34,36,41,46], there was a surprising lack of interventions that aimed to improve the self-management of nonmotor symptoms. Given the impact of nonmotor symptoms on people with Parkinson disease, this is an important gap that should be further investigated and addressed.

Another key area for future research would be the cost-effectiveness of self-management interventions. Only 1 of the included studies examined cost-effectiveness, which also identified a lack of the resources needed to conduct high-quality, cost-effectiveness evaluations (eg, the lack of a specific “cost-of-illness” questionnaire for people with Parkinson disease) [46]. This will be an important area to explore, as several of the interventions appeared to be resource-intensive, especially the interventions that involved home visits by therapists. Although there is likely to be an offset of costs if these interventions improve the quality of life, slow the deterioration of health, and reduce the need for expensive treatments, these interventions will need to be rigorously evaluated to demonstrate the potential benefit of their widespread adoption.

### Conclusion

This scoping review aimed to examine and provide an overview of the state of the literature on self-management interventions for people with Parkinson disease. There is a large amount of research in this area, including several RCTs, that focus on a variety of types of self-management intervention. Most of the studies reported at least some evidence of effectiveness or positive effect of the intervention examined, with the best evidence of effectiveness coming from the RCTs. However, the majority of the studies reviewed focused on motor-related interventions and outcomes, with few interventions aimed at addressing the nonmotor aspects of Parkinson disease. There was also an apparent lack of consideration of the cost-effectiveness of the interventions. Further research will be needed to compare the potential health and economic benefits of implementing interventions to support self-management in people with Parkinson disease with the costs of delivering the interventions. Although some of the studies examined interventions that used digital technologies to monitor symptoms or provide feedback, many of the interventions had substantial in-person time commitments. Future investigations could compare the effectiveness of delivering interventions in person or through digital technologies to potentially improve the cost-effectiveness and availability of self-management support.

## Appendix

Multimedia Appendix 1 PRISMA-ScR (Preferred Reporting Items for Systematic Reviews and Meta-Analyses Extension for Scoping Reviews ) checklist.

Multimedia Appendix 2 Search record.

Multimedia Appendix 3 EndNote search criteria.

Multimedia Appendix 4 Data extraction table.

## Abbreviations

AUC: area under the receiving operator curve
CP: care partner
MeSH: Medical Subject Headings
NHS: National Health Service
PICOS: Population, Intervention, Comparator, Outcome, and Study
PRISMA-ScR: Preferred Reporting Items for Systematic Reviews and Meta-Analyses Extension for Scoping Reviews
RCT: randomized controlled trial

## References

[1] Yang W, Hamilton JL, Kopil C, Beck JC, Tanner CM, Albin RL, Ray Dorsey E, Dahodwala N, Cintina I, Hogan P, Thompson T (2020). Current and projected future economic burden of Parkinson's disease in the U.S. NPJ Parkinsons Dis.

[2] Weir S, Samnaliev M, Kuo T, Tierney TS, Walleser Autiero S, Taylor RS, Schrag A (2018). Short- and long-term cost and utilization of health care resources in Parkinson's disease in the UK. Mov Disord.

[3] (2018). The incidence and prevalence of Parkinson's in the UK: results from the Clinical Practice Research Datalink Summary report. Parkinson's UK.

[4] (2019). NHS Long Term Plan. NHS.

[5] Panagioti M, Richardson G, Small N, Murray E, Rogers A, Kennedy A, Newman S, Bower P (2014). Self-management support interventions to reduce health care utilisation without compromising outcomes: a systematic review and meta-analysis. BMC Health Serv Res.

[6] Feng S, Mäntymäki Matti, Dhir A, Salmela H (2021). How self-tracking and the quantified self promote health and well-being: systematic review. J Med Internet Res.

[7] Hibbard Judith, Gilburt Helen (2014). Supporting people to manage their health: an introduction to patient activation. The King's Fund.

[8] Kessler D, Hauteclocque J, Grimes D, Mestre T, Côtéd Diane, Liddy C (2019). Development of the Integrated Parkinson's Care Network (IPCN): using co-design to plan collaborative care for people with Parkinson's disease. Qual Life Res.

[9] Chaudhuri KR, Prieto-Jurcynska C, Naidu Y, Mitra T, Frades-Payo B, Tluk S, Ruessmann A, Odin P, Macphee G, Stocchi F, Ondo W, Sethi K, Schapira A, Martinez Castrillo Juan Carlos, Martinez-Martin P (2010). The nondeclaration of nonmotor symptoms of Parkinson's disease to health care professionals: an international study using the nonmotor symptoms questionnaire. Mov Disord.

[10] Non-motor symptoms of Parkinson's. Parkinson's UK.

[11] Marinus J, Zhu K, Marras C, Aarsland D, van Hilten JJ (2018). Risk factors for non-motor symptoms in Parkinson's disease. Lancet Neurol.

[12] Minar M, Dragasek J, Valkovic P (2019). Comorbidities in Parkinson's disease – the results from national epidemiological study cosmos. J Neurol Sci.

[13] Findley LJ (2007). The economic impact of Parkinson's disease. Parkinsonism Relat Disord.

[14] Gumber A, Ramaswamy B, Thongchundee O (2019). Effects of Parkinson's on employment, cost of care, and quality of life of people with condition and family caregivers in the UK: a systematic literature review. Patient Relat Outcome Meas.

[15] (2012). Helping people share decision making. The Health Foundation.

[16] Lin CE, Wood JJ, Volkmar FR (2013). Self-management interventions. Encyclopedia of Autism Spectrum Disorders.

[17] Jonkman NH, Schuurmans MJ, Jaarsma T, Shortridge-Baggett LM, Hoes AW, Trappenburg JCA (2016). Self-management interventions: proposal and validation of a new operational definition. J Clin Epidemiol.

[18] Morgan C, Rolinski M, McNaney R, Jones B, Rochester L, Maetzler W, Craddock I, Whone AL (2020). Systematic review looking at the use of technology to measure free-living symptom and activity outcomes in Parkinson's disease in the home or a home-like environment. J Parkinsons Dis.

[19] Channa A, Popescu N, Ciobanu V (2020). Wearable solutions for patients with Parkinson's disease and neurocognitive disorder: a systematic review. Sensors (Basel).

[20] Deb R, Bhat G, An S, Shill H, Ogras U Trends in technology usage for Parkinson’s disease assessment: a systematic review. medRxiv.

[21] Vaartio-Rajalin Heli, Rauhala Auvo, Fagerström Lisbeth (2019). Person-centered home-based rehabilitation for persons with Parkinson's disease: a scoping review. Int J Nurs Stud.

[22] Tuijt R, Tan A, Armstrong M, Pigott J, Read J, Davies N, Walters K, Schrag A (2020). Self-management components as experienced by people with Parkinson's disease and their carers: a systematic review and synthesis of the qualitative literature. Parkinsons Dis.

[23] Kessler D, Liddy C (2017). Self-management support programs for persons with Parkinson's disease: an integrative review. Patient Educ Couns.

[24] Kane E, Schrag A, Walters K, Pigott J (2019). Self-management interventions in Parkinson's disease: a systematic review and meta-analysis. PROSPERO.

[25] Steinhubl SR, Muse ED, Topol EJ (2015). The emerging field of mobile health. Sci Transl Med.

[26] Wannheden C, Revenäs Åsa (2020). How people with Parkinson's disease and health care professionals wish to partner in care using eHealth: co-design study. J Med Internet Res.

[27] Tricco AC, Lillie E, Zarin W, O'Brien KK, Colquhoun H, Levac D, Moher D, Peters MDJ, Horsley T, Weeks L, Hempel S, Akl EA, Chang C, McGowan J, Stewart L, Hartling L, Aldcroft A, Wilson MG, Garritty C, Lewin S, Godfrey CM, Macdonald MT, Langlois EV, Soares-Weiser K, Moriarty J, Clifford T, Tunçalp Özge, Straus SE (2018). PRISMA Extension for Scoping Reviews (PRISMA-ScR): checklist and explanation. Ann Intern Med.

[28] Chivers Seymour K, Pickering R, Rochester L, Roberts HC, Ballinger C, Hulbert S, Kunkel D, Marian IR, Fitton C, McIntosh E, Goodwin VA, Nieuwboer A, Lamb SE, Ashburn A (2019). Multicentre, randomised controlled trial of PDSAFE, a physiotherapist-delivered fall prevention programme for people with Parkinson's. J Neurol Neurosurg Psychiatry.

[29] Connor KI, Cheng EM, Barry F, Siebens HC, Lee ML, Ganz DA, Mittman BS, Connor MK, Edwards LK, McGowan MG, Vickrey BG (2019). Randomized trial of care management to improve Parkinson disease care quality. Neurology.

[30] Ashburn A, Fazakarley L, Ballinger C, Pickering R, McLellan LD, Fitton C (2007). A randomised controlled trial of a home based exercise programme to reduce the risk of falling among people with Parkinson's disease. J Neurol Neurosurg Psychiatry.

[31] Ashburn A, Pickering R, McIntosh E, Hulbert S, Rochester L, Roberts HC, Nieuwboer A, Kunkel D, Goodwin VA, Lamb SE, Ballinger C, Seymour KC (2019). Exercise- and strategy-based physiotherapy-delivered intervention for preventing repeat falls in people with Parkinson's: the PDSAFE RCT. Health Technol Assess.

[32] Collett J, Franssen M, Meaney A, Wade D, Izadi H, Tims M, Winward C, Bogdanovic M, Farmer A, Dawes H (2017). Phase II randomised controlled trial of a 6-month self-managed community exercise programme for people with Parkinson's disease. J Neurol Neurosurg Psychiatry.

[33] Collett J, Franssen M, Winward C, Izadi H, Meaney A, Mahmoud W, Bogdanovic M, Tims M, Wade D, Dawes H (2017). A long-term self-managed handwriting intervention for people with Parkinson's disease: results from the control group of a phase II randomized controlled trial. Clin Rehabil.

[34] Sturkenboom IHWM, Graff MJL, Hendriks JCM, Veenhuizen Y, Munneke M, Bloem BR, Nijhuis-van der Sanden MW, OTiP study group (2014). Efficacy of occupational therapy for patients with Parkinson's disease: a randomised controlled trial. Lancet Neurol.

[35] Tickle-Degnen L, Ellis T, Saint-Hilaire MH, Thomas CA, Wagenaar RC (2010). Self-management rehabilitation and health-related quality of life in Parkinson's disease: a randomized controlled trial. Mov Disord.

[36] van der Kolk NM, de Vries NM, Kessels RPC, Joosten H, Zwinderman AH, Post B, Bloem BR (2019). Effectiveness of home-based and remotely supervised aerobic exercise in Parkinson's disease: a double-blind, randomised controlled trial. Lancet Neurol.

[37] Arroyo-Gallego T, Ledesma-Carbayo MJ, Sanchez-Ferro A, Butterworth I, Mendoza CS, Matarazzo M, Montero P, Lopez-Blanco R, Puertas-Martin V, Trincado R, Giancardo L (2017). Detection of motor impairment in Parkinson's disease via mobile touchscreen typing. IEEE Trans Biomed Eng.

[38] Arroyo-Gallego T, Ledesma-Carbayo MJ, Butterworth I, Matarazzo M, Montero-Escribano P, Puertas-Martín Verónica, Gray ML, Giancardo L, Sánchez-Ferro Álvaro (2018). Detecting motor impairment in early Parkinson's disease via natural typing interaction with keyboards: validation of the neuroQWERTY approach in an uncontrolled at-home setting. J Med Internet Res.

[39] Ferraris C, Nerino R, Chimienti A, Pettiti G, Cau N, Cimolin V, Azzaro C, Albani G, Priano L, Mauro A (2018). A self-managed system for automated assessment of UPDRS upper limb tasks in Parkinson's disease. Sensors (Basel).

[40] Butterfield LC, Cimino CR, Salazar R, Sanchez-Ramos J, Bowers D, Okun MS (2017). The Parkinson's Active Living (PAL) program. J Geriatr Psychiatry Neurol.

[41] Pretzer-Aboff I, Galik E, Resnick B (2011). Feasibility and impact of a function focused care intervention for Parkinson's disease in the community. Nurs Res.

[42] Chomiak T, Watts A, Meyer N, Pereira FV, Hu B (2017). A training approach to improve stepping automaticity while dual-tasking in Parkinson's disease: a prospective pilot study. Medicine (Baltimore).

[43] Dobkin RD, Interian A, Durland JL, Gara MA, Menza MA (2018). Personalized telemedicine for depression in Parkinson's disease: a pilot trial. J Geriatr Psychiatry Neurol.

[44] Fisher JM, Hammerla NY, Rochester L, Andras P, Walker RW (2016). Body-worn sensors in Parkinson's disease: evaluating their acceptability to patients. Telemed J E Health.

[45] Connor KI, Siebens HC, Mittman BS, McNeese-Smith DK, Ganz DA, Barry F, Edwards LK, McGowan MG, Cheng EM, Vickrey BG (2020). Stakeholder perceptions of components of a Parkinson disease care management intervention, care coordination for health promotion and activities in Parkinson's disease (CHAPS). BMC Neurol.

[46] Cubo E, Mariscal N, Solano B, Becerra V, Armesto D, Calvo S, Arribas J, Seco J, Martinez A, Zorrilla L, Heldman D (2017). Prospective study on cost-effectiveness of home-based motor assessment in Parkinson's disease. J Telemed Telecare.

[47] Pickering RM, Fitton C, Ballinger C, Fazakarley L, Ashburn A (2013). Self reported adherence to a home-based exercise programme among people with Parkinson's disease. Parkinsonism Relat Disord.

[48] Mandrekar JN (2010). Receiver operating characteristic curve in diagnostic test assessment. J Thorac Oncol.

[49] Todorova A, Jenner P, Ray Chaudhuri K (2014). Non-motor Parkinson's: integral to motor Parkinson's, yet often neglected. Pract Neurol.

[50] Hermanowicz N, Jones SA, Hauser RA (2019). Impact of non-motor symptoms in Parkinson's disease: a PMDAlliance survey. Neuropsychiatr Dis Treat.

[51] Gökçal Elif, Gür Veysel Eren, Selvitop R, Babacan Yildiz G, Asil T (2017). Motor and non-motor symptoms in Parkinson's disease: effects on quality of life. Noro Psikiyatr Ars.

[52] Pfeiffer RF (2016). Non-motor symptoms in Parkinson's disease. Parkinsonism Relat Disord.

