# Supplementary material for: Self-management Interventions for People With Parkinson Disease: Scoping Review
Source: J Med Internet Res. 2022 Aug 5;24(8):e40181. doi: 10.2196/40181 (PMC9391969; doi:10.2196/40181)
Supplement: Multimedia Appendix 2 [file jmir_v24i8e40181_app2.docx]

### Multimedia Appendix 2. Search record

| **Database** | **Search String** | **Retrieved** |
| --- | --- | --- |
| PubMed | ((Parkinson Disease[MeSH Terms]) AND (Self-Management OR Self-Care OR Home Nursing OR Delivery of Health Care, Integrated OR Telemedicine OR Mobile Applications OR Internet-based Interventions OR Internet of Things[MeSH Terms])) AND (Self Efficacy OR Quality of Life OR Signs and Symptoms OR Health Behaviour OR Patient Admission OR Patient Readmission[MeSH Terms]) | 511 |
| Ovid | (Parkinson Disease/) AND (Self-Management/ or Self Care/ or Home Nursing/ or “Delivery of Health Care, Integrated”/ or Telemedicine/ or Mobile Applications/ or Internet-based Intervention/ or “Internet of Things”/) AND (Self-Efficacy/ or “Quality of Life”/ or symptoms.ti,ab or Health Behavior/ or Patient Admission/ or Patient Readmission/) | 590 |
| Scopus | TITLE-ABS-KEY(("Parkinson* Disease") AND (Self-Management OR Self-Care OR "Home Nursing" OR "integrated Delivery of Health Care" OR Telemedicine OR "Mobile Applications" OR "Internet-based Interventions" OR "Internet of Things") AND ("Self Efficacy" OR "Quality of Life" OR Symptoms OR "Health Behaviour" OR "Patient Admission" OR "Patient Readmission")) | 403 |
| Web of Science | TOPIC^a^: ("Parkinson* Disease") *AND* TOPIC: (Self-Management OR Self-Care OR "Home Nursing" OR "integrated Delivery of Health Care" OR Telemedicine OR "Mobile Applications" OR "Internet-based Interventions" OR "Internet of Things") *AND* TOPIC: ("Self Efficacy" OR "Quality of Life" OR Symptoms OR "Health Behaviour" OR "Patient Admission" OR "Patient Readmission") | 79 |

^a^TOPIC searches the title, abstract, author keywords, and Keywords Plus

### 
