# Supplementary material for: Self-management Interventions for People With Parkinson Disease: Scoping Review
Source: J Med Internet Res. 2022 Aug 5;24(8):e40181. doi: 10.2196/40181 (PMC9391969; doi:10.2196/40181)
Supplement: Multimedia Appendix 3 [file jmir_v24i8e40181_app3.docx]

### Multimedia Appendix 3: Endnote search criteria

| **Pass^a^** | **Search string** | **# of references remaining** |
| --- | --- | --- |
| 1 | Title = Parkinson’s | 816 |
| 2 | Abstract = self manag* OR self care OR home OR mHealth OR intervention OR remote monitor* OR program* OR care delivery OR care pathway | 236 |
| 3 | Abstract = case-control OR case control OR randomised control* OR randomized control* OR RCT OR cohort | 63 |
| 4 | Title = NOT (protocol OR review) | 51 |

^a^Each pass was conducted on the subset of studies retrieved in the previous pass.
